# Post-transplant recurrent pericarditis with pericardial tamponade is successfully treated with colchicine: A case report

**DOI:** 10.3892/etm.2014.1824

**Published:** 2014-07-04

**Authors:** CHEN GAO, FENGHUA PENG, LONGKAI PENG

**Affiliations:** Department of Urological Organ Transplantation, The Second Xiangya Hospital of Central South University, Changsha, Hunan 410011, P.R. China

**Keywords:** kidney transplant, recurrent pericarditis, pericardial tamponade, colchicine

## Abstract

Recurrent pericarditis is a rare complication following renal transplantation. Colchicine, an inhibitor of microtubule polymerization, has been recommended for the treatment of recurrent acute pericarditis in non-transplant patients and is commonly used for the treatment of gout in transplant patients. However, the use of colchicine for the treatment of recurrent pericarditis in renal transplant patients has rarely been reported. In the present study, a rare case of recurrent pericarditis, manifested as large pericardial effusion and pericardial tamponade within the first year following renal transplantation, was successfully treated with colchicine. Therefore, low-dose colchicine may be a safe and effective option for the treatment of recurrent pericarditis in renal transplant patients.

## Introduction

Pericarditis is an uncommon complication following renal transplantation, with a reported incidence of 2.4% in the first two months following renal transplantation (1). Multiple factors may contribute to the development of this disorder, including catabolism abnormalities secondary to surgery and drugs and an increased risk of infections due to immunosuppressive therapy (1,2). Pericardial tamponade, which is caused by effusion accumulation and increased intrapericardial pressure, is a severe complication of pericarditis (3). Therapeutic strategies targeting pericarditis should focus on the etiology as much as possible. However, when the etiology is unclear or idiopathic, and inflammatory markers are elevated, nonsteroidal anti-inflammatory drugs, colchicine and corticosteroids are the most common therapeutic modalities for non-transplant patients (4). The current study presents a rare case of recurrent pericarditis that manifested as large pericardial effusion and pericardial tamponade within the first year following kidney transplantation. The condition was successfully treated with colchicine. To the best of our knowledge, the use of colchicine for the treatment of recurrent pericarditis in kidney transplant patients has rarely been reported.

## Case report

A 31-year-old male with end-stage renal disease, secondary to chronic glomerulonephritis (IgA nephropathy), received a kidney transplant in December 2010 from a 25-year-old male donor who succumbed to cardiac death as a result of craniocerebral trauma. Written informed consent for inclusion in the case report was provided by the patient’s family. The patient had been maintained on hemodialysis for one year prior to the transplantation and had a history of tuberculosis contact and digestive tract hemorrhage. Preoperative chest X-rays, electrocardiograms and echocardiography examinations revealed no abnormalities. Cytomegalovirus and hepatitis B and C viruses were seronegative in the donor and recipient. Surgical follow-up was uncomplicated. The immunosuppressive regimen included 0.5 g intravenous methylprednisolone during surgery and 0.5 g/day for the first three days following the transplantation. Tacrolimus was administered according to the patient’s body weight. As the body weight was 65 kg, tacrolimus was administered at 6.5 mg/day for the initial three days. Subsequently, the dose was adjusted according to the trough concentration of tacrolimus, and 6–8 ng/ml tacrolimus was applied for the first six months following transplantation and 5–6 ng/ml was administered for months 6–12 following transplantation. Mycophenolate mofetil (0.75 g) was administered orally every 12 h, which was gradually reduced to 0.5 g per 12 h for maintenance. Furthermore, prednisolone at a first dose of 80 mg/day was applied, which was gradually tapered during a six-month-period to 10 mg/day for long-term maintenance. The allograft function was excellent immediately following surgery.

**Figure 1:**
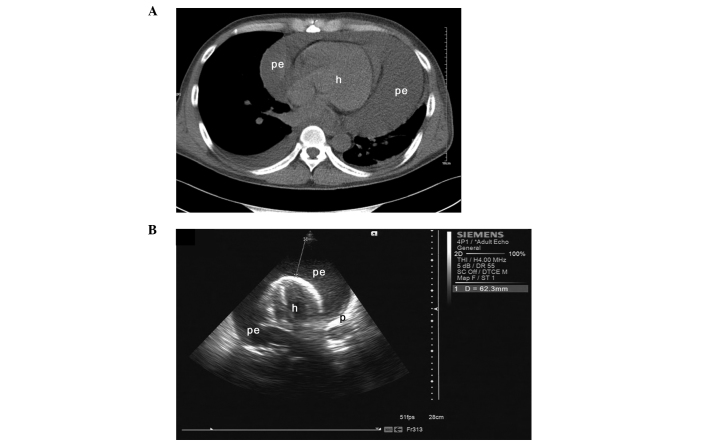
(A) Chest computed tomography scan revealed an abundant circumferential pericardial effusion and pleural effusion. (B) Echocardiography scan revealed an abundant circumferential pericardial effusion with a maximal echo-free space of 62.3 mm. h, heart; pe, pericardial effusion; p, pericardium.

However, at day 11 following the transplantation, the patient developed symptoms of chest pain and dyspnea, and a decrease in urinary volume (from >2,000 to 1,000 ml/day) was observed. A physical examination revealed tachycardia, a decrease in systolic pressure and jugular venous distention. Echocardiography and chest computed tomography scans revealed an abundant compressive circumferential pericardial effusion (echo-free space, 62.3 mm) and pleural effusion (Fig. 1) with 49% of the left ventricular ejection fraction. Pericardiocentesis was performed and a lemon-colored exudative fluid (3,000 ml; protein level, 51.4 g/l; fluid/serum ratio, >0.5) with abundant polymorphonuclear cells was removed, which relieved the patients symptoms. Further investigations revealed that the erythrocyte sedimentation rate was 6 mm/h, the serum creatinine level was 200 μmol/l, the serum lactate dehydrogenase level was 339.3 U/l (serum/fluid ratio, >0.6) and the adenosine deaminase level in the pericardial fluid was 3.5 U/l. Negative results were observed for serum anti-nuclear antibodies and tumor markers, including carcinoembryonic antigen, carbohydrate antigen (CA) 125, CA 15-3 and CA 19-9. Serological tests for cytomegalovirus, Epstein-Barr virus, mycoplasma, chlamydia, coxsackie virus, herpes simplex virus, *Mycobacterium tuberculosis* and toxoplasma were negative. In addition, direct examination of the pericardial fluid for mycobacteria, *Aspergillus* and yeast, as well as a pericardial fluid culture for bacteria, were negative. The drainage was maintained for 12 days. A repeated echocardiogram scan did not detect any pericardial effusion. Thus, the patient was discharged following removal of the drainage tube, and once the serum creatinine level had reached 150 μmol/l.

**Figure 2:**
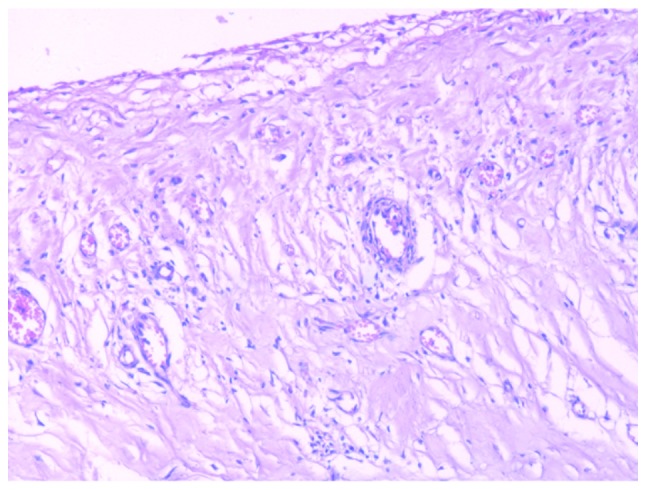
Histopathological examination of the pericardium revealed chronic inflammatory changes, including the infiltration of lymphocytes (hematoxylin-eosin staining; magnification, ×100).

**Figure 3:**
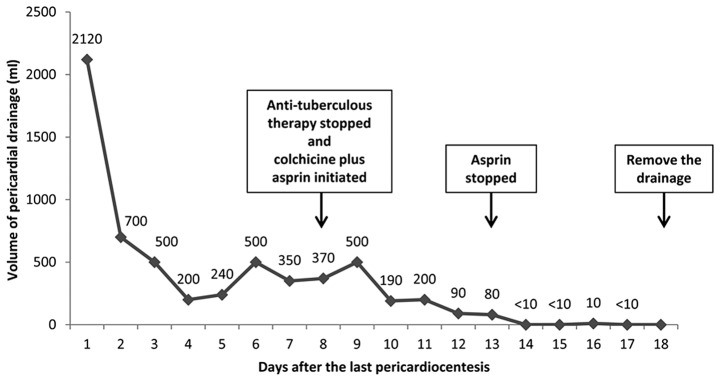
Variation in the volume of the pericardial drainage fluid following the last pericardiocentesis.

However, the patient was readmitted after 20 days with pericardial tamponade, which required pericardiocentesis to be performed. A lemon-colored exudative fluid (600 ml) was removed and a catheter was maintained. The aforementioned etiological examinations were performed again, with all tests negative, with the exception of the enzyme-linked immunospot test (ELISPOT) for *Mycobacterium tuberculosis*. Therefore, at day 48 following transplantation, a two-month period of empirical antituberculous therapy (rifampicin, isoniazid, ethambutol, pyrazinamide and moxifloxacin) was proposed, which was prolonged to three months. However, during this time period, the volume of pericardial drainage fluid remained at 200–600 ml per day. At day 77 following transplantation, a pericardial window with thoracic close drainage was performed due to the frequent obstruction of the catheter. Postoperative pericardial histopathological assessment revealed chronic inflammatory changes without any evidence of malignancy or tuberculosis (Fig. 2). Furthermore, the culture of pericardial fluid tested negative for *Mycobacterium tuberculosis* after 60 days. At day 127 following transplantation, a pericardiocentesis was performed again to replace the blocked thoracic close drainage with a catheter. The ELISPOT was repeated and negative results were achieved. Since no specific causes were identified, including infections or malignancy, and the prolonged (three months) empirical antituberculous therapy failed to ameliorate the symptoms, the patient was diagnosed with idiopathic recurrent pericarditis. Antituberculous therapy was stopped and colchicine (0.5 mg twice daily) plus aspirin (500 mg twice daily, which was stopped after five days due to suspicion of digestive tract hemorrhage) were prescribed at day 135 following transplantation. The effusion regressed rapidly thereafter. At day six following the initiation of colchicine treatment, the volume of the drainage fluid decreased to <10 ml per day. The drainage tubes were removed at day 10 following the initiation of treatment, since minimal residual effusion was observed by echocardiography (Fig. 3). Treatment was maintained for 12 months with transient reversible elevation of alanine aminotransferase (peak value, 80 U/l). Within the 30 months of follow-up since the treatment was initiated, no relapse has been observed. In addition, the serum creatinine levels have remained between 130 and 145 μmol/l.

## Discussion

Pericarditis is a common complication in patients with end-stage renal disease, although the incidence rate decreases significantly following renal transplantation (1,3). In a study of 1,497 patients by Sever *et al* (1), it was demonstrated that during the first two months following renal transplantation, the etiologies of acute pericarditis included uremia, bacterial or viral infection, idiopathy and drugs (1). Among the etiologies, tuberculosis was the most common in developing countries (4). In the present case, extensive investigation into the etiology was performed to exclude the majority of the potential causes, including infection and malignancy. In particular, a prolonged three-month period of empirical antituberculous therapy plus culture of pericardial fluid for *Mycobacterium tuberculosis* were performed on the basis of the positive result from the ELISPOT and the epidemiological background of the patient to exclude a potential tuberculous infection. Uremic pericarditis is not uncommon in renal transplant patients, particularly among those with insufficient allograft function (1); however, the condition should regress along with the recovery of allograft function. A number of drugs, including sirolimus and minoxidil, have been reported to induce pericardial effusion and even pericardial tamponade in renal transplant patients (1,5). However, these drugs were not administered to the patient in the present study. The pathogenesis of the idiopathic pericarditis remains complicated and obscure. In certain cases, undiagnosed viral infections may contribute to the development of the disorder (6). Furthermore, due to the limitation of the etiological tests, viral causes are unable to be excluded in the present case study.

Acute pericarditis may recur in up to 50% of patients following the first incidence (7), and is severe complication. Currently, available therapeutic treatments include nonsteroidal anti-inflammatory drugs (NSAID), colchicine and corticosteroid. Colchicine, an inhibitor of microtubule polymerization, exerts anti-inflammatory effects by inhibiting the migration and activities of polymorphonuclear cells. The drug has been approved for the treatment of several inflammatory diseases, including familial Mediterranean fever, gout and Behcet’s disease (8). Previous studies have also demonstrated that colchicine, applied alone or in combination with conventional therapies, such as NSAIDs, may significantly reduce the recurrence rate, the persistence of the symptoms and the mean number of recurrences, as well as prolong the time to subsequent recurrence (9,10). Despite results from additional studies being contradictory, corticosteroids are commonly used for the treatment of recurrent pericarditis (11). However, an international multicenter study proposed that pretreatment with corticosteroids impeded the efficacy of colchicine and was associated with a higher percentage of relapse (12). In the present case, as immunosuppression was required, immunosuppressive regimens, including corticosteroids (10 mg/day), were maintained during and following colchicine therapy. No relapse occurred following the initiation of colchicine treatment. The curative effects of colchicine are controversial due to the previous administration of antituberculous therapy. However, during the 30 months follow-up, no evidence of tuberculosis was observed in the patient, although it is known that a three-month-period of antituberculous therapy is not sufficient for curing tuberculosis.

The use of colchicine in transplant patients is limited and cautious due to the toxicity. Previous studies have demonstrated that colchicine induces severe rhabdomyolysis in solid organ transplant patients when used for the treatment of acute gout arthritis (13,14). The causes may be attributed to the concurrent use of P-glycoprotein inhibitors, such as cyclosporin, pravastatin and azithromycin, high doses or impaired renal or hepatic functions (13,14). However, in the present case, during the 12-month period of colchicine treatment, only modest transient reversible side effects were observed and the allograft function remained stable. A recent study demonstrated that tacrolimus was also an inhibitor of P-glycoprotein; however, less active than cyclosporine (15). Thus, when administered in combination, tacrolimus may reduce the likelihood of severe colchicine intoxication in renal transplant patients. However, further investigation is required.

In conclusion, the current study presented a rare case of recurrent pericarditis and pericardial tamponade within one year of renal transplantation, which was successfully treated with colchicine. Therefore, low-dose colchicine may be a safe and effective strategy for the treatment of recurrent pericarditis in renal transplant patients.
